# Interaction between severe postoperative complications and adjuvant chemotherapy on overall survival after upfront resection of pancreatic ductal adenocarcinoma

**DOI:** 10.3389/fmed.2026.1968161

**Published:** 2026-10-07

**Authors:** Shiwei Wu, Daobin Wang, Dongxing Zhang, Jiayue Zou, Xiaoyuan Hu, Jun He, Xiaofeng Xue, Lei Qin

**Affiliations:** 1 Pancreatic Disease Center, The First Affiliated Hospital of Soochow University, Suzhou, Jiangsu, China; 2 Department of General Surgery, The First Affiliated Hospital of Soochow University, Suzhou, Jiangsu, China

**Keywords:** adjuvant chemotherapy, Clavien-Dindo classification, interaction, pancreatic ductal adenocarcinoma, postoperative complications, severe complications, stratified cox regression

## Abstract

**Objective:**

Severe postoperative complications (Clavien-Dindo grade ≥3) occur in 10%–30% of patients after curative resection of pancreatic ductal adenocarcinoma (PDAC). Whether adjuvant chemotherapy modifies their impact on long-term survival remains unclear. This study aimed to systematically evaluate the interaction between severe complications and adjuvant chemotherapy after PDAC resection.

**Methods:**

We retrospectively analyzed 383 patients with pathologically confirmed PDAC who underwent upfront curative-intent resection at our center between January 2018 and January 2024. Our institutional database initially contained 398 surgical records; 15 records meeting the R2 margin-status or M1-metastasis exclusion criteria were excluded, leaving 383 patients for the primary analysis. Median overall survival (OS) was estimated using the Kaplan–Meier method, and group comparisons were performed with the log-rank test. Covariates adjusted for in the multivariable Cox regression model included age, sex, BMI, tumor differentiation, margin status, vascular invasion, perineural invasion, lymph node ratio, and tumor size. Stratified Cox regression and interaction-effect models were further constructed. Exploratory recurrence-free survival (RFS) analyses defined an event as recurrence or death from any cause. Sensitivity analyses excluded patients with OS <60 days and, additionally, evaluated a 90-day threshold.

**Results:**

Severe complications occurred in 40 of 383 patients (10.4%). Adjuvant chemotherapy was received by 16 of 40 patients (40.0%) in the severe-complication group and 205 of 343 patients (59.8%) in the non-severe-complication group (*P* = 0.026). Among patients who did not receive chemotherapy, median OS was significantly shorter in the severe-complication group than in the non-severe-complication group (7.9 vs. 14.6 months, *P* < 0.001); among those who received chemotherapy, the difference was not statistically significant (18.8 vs. 20.5 months, *P* = 0.445). In the interaction model, severe complications were associated with increased mortality in the no-chemotherapy stratum (HR: 4.18, 95% CI: 2.49–7.01, *P* < 0.001) but not in the chemotherapy stratum (HR: 1.24, 95% CI: 0.66–2.34, *P* = 0.511). In separate stratified Cox models adjusted for the same covariates, adjuvant chemotherapy was associated with lower mortality in the severe-complication group (HR: 0.15, 95% CI: 0.04–0.57, *P* = 0.005), whereas the association was not statistically significant in the non-severe-complication group (HR: 0.76, 95% CI: 0.56–1.03, *P* = 0.075). Exploratory RFS analysis showed shorter RFS among patients with severe complications (median 6.4 vs. 11.1 months; adjusted HR: 1.58, 95% CI: 1.09–2.28, *P* = 0.016).

**Conclusions:**

In this retrospective cohort, severe complications were associated with worse OS among patients who did not receive adjuvant chemotherapy, whereas the association was not statistically significant among those who received chemotherapy. Because the severe-complication subgroup was small and treatment receipt was subject to postoperative selection, the interaction should be considered hypothesis-generating. These findings do not establish that adjuvant chemotherapy offsets the adverse prognostic association of severe complications and require prospective validation before informing treatment decisions.

## 1 Introduction

Pancreatic ductal adenocarcinoma (PDAC) has the poorest prognosis among digestive tract malignancies, with a five-year survival rate of approximately 13% and rising incidence and mortality in both the United States and China (1). Curative surgical resection remains the only potentially curative treatment; however, even after successful R0 resection, the five-year survival rate is only 20%–25% (2). To improve long-term outcomes, adjuvant chemotherapy has been established as the standard of care (3, 4). The PRODIGE 24 trial demonstrated that mFOLFIRINOX significantly prolonged median OS compared with gemcitabine (54.4 vs. 35.0 months) (5); the JASPAC 01 trial confirmed the superiority of S-1 over gemcitabine in Japanese patients (6); and the ESPAC-4 trial showed that gemcitabine plus capecitabine was superior to gemcitabine alone (7).

Pancreatic surgery, however, is associated with a high complication rate (8). Intra-abdominal infection, postoperative hemorrhage, and delayed gastric emptying are among the common postoperative events. According to the Clavien-Dindo classification, severe complications (grade ≥3) require endoscopic, interventional, or surgical management (9). Accumulating evidence indicates that postoperative complications after pancreatic cancer surgery are associated not only with increased perioperative risk but also with worse long-term survival (10–15). Two principal mechanisms have been proposed to explain this association. First, surgical trauma and persistent postoperative inflammation can induce systemic immunosuppression, for example, through expansion of myeloid-derived suppressor cells (MDSCs) and activation of the NOX2/ROS axis, thereby creating an immune-evasive microenvironment for minimal residual disease (16, 17). Second, severe complications often delay postoperative recovery and hinder the administration of adjuvant chemotherapy; patients who develop complications within 30 days after surgery are significantly less likely to receive adjuvant chemotherapy (OR: 0.59) (11, 15, 18–20). Notably, previous studies have generally treated severe complications and adjuvant chemotherapy as independent prognostic factors or have examined the latter solely as a mediator of the former's effect on survival (15, 21, 22).

However, a more fundamental scientific question has been largely overlooked: can the adverse prognostic effect of severe complications be modified by adjuvant chemotherapy? In other words, among patients who ultimately receive adjuvant chemotherapy, does the excess mortality risk conferred by severe complications persist? Or can chemotherapy, through direct eradication of micrometastases or reversal of the immunosuppressive state, offset the complication-driven pro-tumorigenic effect? To our knowledge, the interaction between severe postoperative complications and adjuvant chemotherapy has not been well characterized in relation to long-term survival.

To address this knowledge gap, we conducted a retrospective cohort study employing stratified Cox regression and interaction-effect models to systematically evaluate the interplay between severe complications and adjuvant chemotherapy after curative-intent PDAC resection, with the aim of clarifying whether adjuvant chemotherapy modifies the prognostic impact of severe complications and providing evidence for individualized postoperative treatment strategies.

## 2 Methods

### 2.1 Study design and population

This was a retrospective cohort study of patients with upfront-resected PDAC. Our institutional database contained 398 surgical records with unique inpatient IDs. Patients with an R2 margin status or intraoperative detection of M1 metastasis were excluded; 15 records were excluded after application of these criteria, leaving a final analytic cohort of 383 patients who underwent curative-intent pancreatectomy at our center between January 2018 and January 2024. Inclusion criteria were: (1) pathologically confirmed PDAC; (2) R0 or R1 margin status after resection with curative intent; and (3) age ≥18 years. Exclusion criteria were: (1) R2 margin status or intraoperative detection of M1 metastasis; (2) preoperative neoadjuvant therapy; and (3) missing data required for the primary OS and RFS analysis (Figure 1).

**Figure 1:**
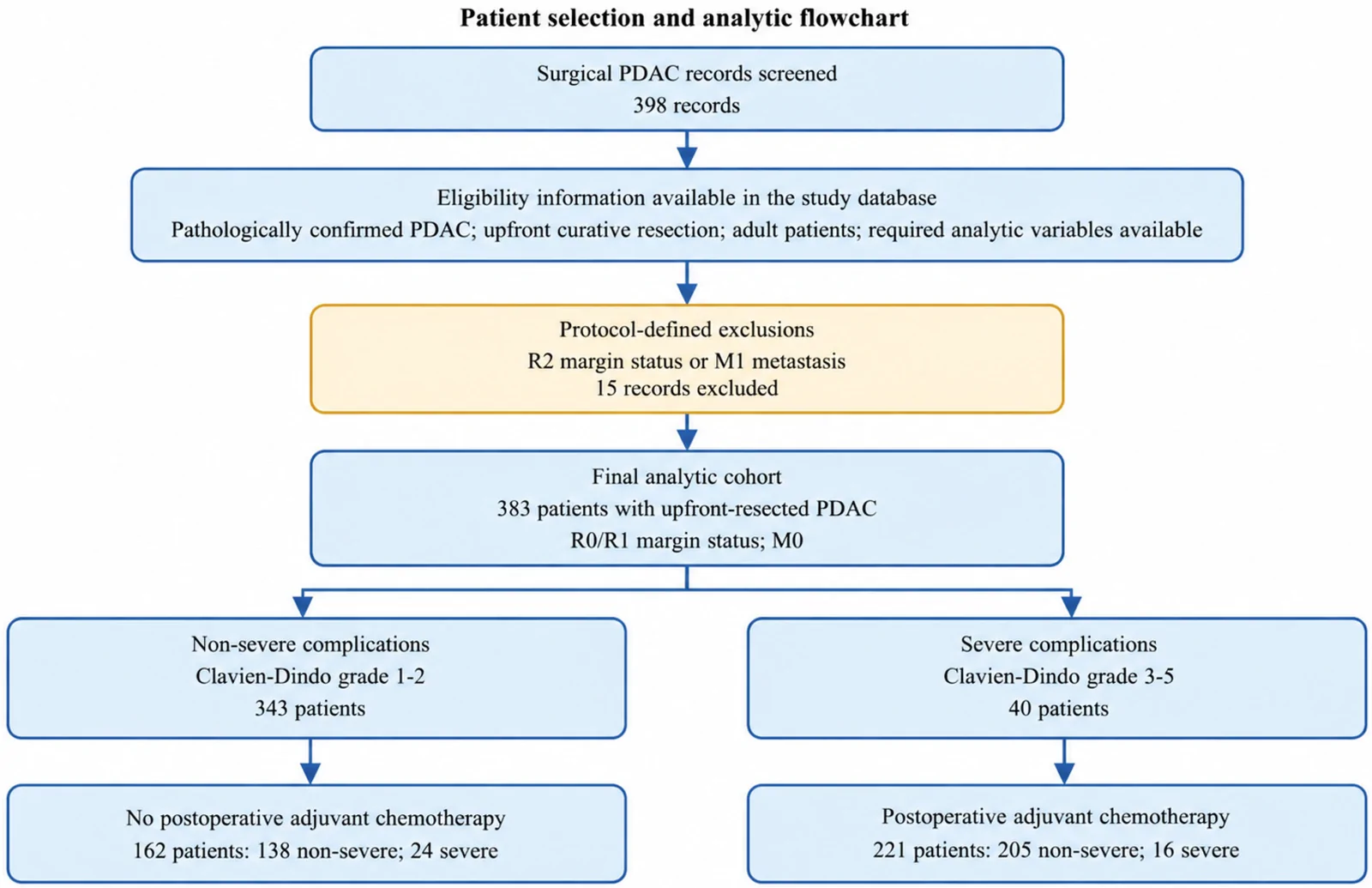
Study flowchart of cohort selection and analytic groups.

### 2.2 Data collection and covariates

The following variables were extracted from electronic medical records: age, sex, BMI calculated from recorded weight and height, history of diabetes, history of cardiovascular disease, tumor differentiation, margin status, vascular invasion, perineural invasion, tumor size, and lymph node ratio (LNR). Adjuvant-treatment data included a treatment indicator, a free-text treatment field, first-chemotherapy date, and surgery-to-chemotherapy interval. Free-text regimen information, first-chemotherapy date, and interval were available for all 221 chemotherapy recipients. For descriptive reporting, the first documented systemic regimen was classified as AG, GS, S-1, gemcitabine/capecitabine, GemOx, FOLFIRINOX, or other; Completed adjuvant cycles, dose intensity, and reasons for not receiving chemotherapy were not available as structured, analyzable variables.

### 2.3 Exposure definitions

Severe complications were defined as Clavien-Dindo grade ≥3 complications occurring within 30 days after surgery. Adjuvant chemotherapy was defined as receipt of postoperative chemotherapy documented in the medical record. The documented regimens included gemcitabine plus nab-paclitaxel (AG), gemcitabine plus oral S-1 (GS), oral S-1 monotherapy, gemcitabine monotherapy, gemcitabine plus capecitabine, gemcitabine plus oxaliplatin (GemOx), and FOLFIRINOX. Because some records also contained sequential or mixed treatments, particularly after recurrence, regimen-specific comparisons were not prespecified.

### 2.4 Outcome

The primary outcome was overall survival (OS), defined as the interval from the date of surgery to the date of death from any cause or the last follow-up. Recurrence-free survival (RFS) was analyzed as a secondary exploratory outcome and was defined as the interval from surgery to the first documented recurrence, death from any cause, or the last follow-up without recurrence. The single initially blank recurrence field was verified as no recurrence and coded accordingly. Cause-specific mortality and cause-of-death data were not collected in our institutional database. Follow-up data in the institutional database were available through March 2026.

### 2.5 Statistical analysis

Continuous variables were expressed as mean ± standard deviation and categorical variables as frequencies (percentages). Group comparisons were performed using the t-test or Mann–Whitney U test for continuous variables and the *χ*^2^ test or Fisher's exact test for categorical variables. Median OS and RFS were estimated using the Kaplan–Meier method and compared with the log-rank test. RFS events were defined as recurrence or death from any cause. To account for potential confounding by adjuvant chemotherapy, cumulative survival curves for severe complications were plotted with stratification by chemotherapy status. Univariable and multivariable Cox proportional hazards regression models were used to identify independent predictors of OS, with covariates forced into the multivariable model including age, sex, BMI, tumor differentiation, margin status, vascular invasion, perineural invasion, tumor size, and lymph node ratio. Stratified Cox regression was performed to estimate the hazard ratio (HR) of adjuvant chemotherapy separately in the severe-complication and non-severe-complication groups, using the same covariates as the main multivariable model. A product interaction term (severe complications × adjuvant chemotherapy) was introduced into the Cox model to formally test for statistical interaction, and its HR and 95% CI were reported. Exploratory adjusted Cox analyses for RFS used the same covariate set as the main multivariable OS model. Sensitivity analysis was conducted by excluding patients with OS <60 days. An additional descriptive sensitivity analysis used a 90-day threshold. All tests were two-sided; *P* values are reported to three decimal places, with *P* < 0.001 used where applicable, and *P* < 0.05 was considered statistically significant. All statistical analyses were performed using R version 4.4.3.

## 3 Results

### 3.1 Baseline characteristics

The cohort included 383 patients who underwent upfront curative-intent PDAC resection, of whom 213 (55.6%) were male, with a mean age of 65.97 ± 9.11 years and a mean BMI of 22.43 ± 3.09 kg/m^2^. Forty patients (10.4%) developed severe complications (Clavien-Dindo grade ≥ 3). Of these, 28 patients (70.0%) developed clinically relevant postoperative pancreatic fistula (CR-POPF; grade B or C). Adjuvant chemotherapy was received by 16 of 40 patients (40.0%) in the severe-complication group and 205 of 343 patients (59.8%) in the non-severe-complication group (*P* = 0.026). Among the 221 chemotherapy recipients, the first documented regimen was AG in 118 (53.4%), S-1 in 21 (9.5%), GS in 15 (6.8%), gemcitabine/capecitabine in 14 (6.3%), GemOx in 5 (2.3%), FOLFIRINOX in 2 (0.9%), and other regimens in 46 (20.8%). First-chemotherapy date and surgery-to-chemotherapy interval were available for all 221 recipients; the median interval was 57 days (IQR, 44–76). As reported descriptively in Table 1, mean observed OS time was shorter in the severe-complication group than in the non-severe-complication group (442.80 vs. 680.03 days, *P* = 0.005). The primary dataset included 7 observed deaths within 30 days after surgery. All occurred in patients who did not receive chemotherapy, including 6 in the severe-complication group and 1 in the non-severe-complication group.

**Table 1:** Comparison of clinicopathological characteristics and outcomes between groups.

Variable	Level	Overall (*n* = 383)	No severe complications (*n* = 343)	Severe complications (*n* = 40)	*P*
Vital status at last follow-up (%)	Alive	152 (39.7)	143 (41.7)	9 (22.5)	0.029
	Dead	231 (60.3)	200 (58.3)	31 (77.5)	
Recurrence status (%)	No	134 (35.0)	119 (34.7)	15 (37.5)	0.860
	Yes	249 (65.0)	224 (65.3)	25 (62.5)	
Sex (%)	Female	170 (44.4)	153 (44.6)	17 (42.5)	0.932
	Male	213 (55.6)	190 (55.4)	23 (57.5)	
History of diabetes (%)	No	245 (64.0)	218 (63.6)	27 (67.5)	0.751
	Yes	138 (36.0)	125 (36.4)	13 (32.5)	
History of cardiovascular disease (%)	No	206 (53.8)	188 (54.8)	18 (45.0)	0.312
	Yes	177 (46.2)	155 (45.2)	22 (55.0)	
Adjuvant chemotherapy (%)	No	162 (42.3)	138 (40.2)	24 (60.0)	0.026
	Yes	221 (57.7)	205 (59.8)	16 (40.0)	
Differentiation (%)	High-Middle	5 (1.3)	4 (1.2)	1 (2.5)	0.680
	Middle	113 (29.5)	104 (30.3)	9 (22.5)	
	Middle-Low	205 (53.5)	181 (52.8)	24 (60.0)	
	Low-Middle	30 (7.8)	28 (8.2)	2 (5.0)	
	Low	30 (7.8)	26 (7.6)	4 (10.0)	
Margin status (%)	R0	325 (84.9)	292 (85.1)	33 (82.5)	0.837
	R1	58 (15.1)	51 (14.9)	7 (17.5)	
Vascular invasion (%)	No	302 (78.9)	269 (78.4)	33 (82.5)	0.695
	Yes	81 (21.1)	74 (21.6)	7 (17.5)	
Perineural invasion (%)	No	114 (29.8)	103 (30.0)	11 (27.5)	0.882
	Yes	269 (70.2)	240 (70.0)	29 (72.5)	
LNR, mean (SD)		0.13 (0.20)	0.13 (0.20)	0.12 (0.14)	0.733
Observed OS, mean (SD), days		655.25 (502.34)	680.03 (502.00)	442.80 (458.33)	0.005
BMI, mean (SD), kg/m^2^		22.43 (3.09)	22.34 (3.07)	23.28 (3.18)	0.067
Age, mean (SD), years		65.97 (9.11)	65.70 (9.05)	68.33 (9.40)	0.084
Tumor size, mean (SD), cm		3.51 (1.50)	3.47 (1.40)	3.88 (2.16)	0.100

### 3.2 Stratified analysis of severe complications and adjuvant chemotherapy on OS

In the no-chemotherapy group, median OS was 7.9 months among patients with severe complications and 14.6 months among those without severe complications (log-rank *P* < 0.001). In the chemotherapy group, median OS was 18.8 months for patients with severe complications and 20.5 months for those without severe complications (log-rank *P* = 0.445). These stratified comparisons are compatible with effect modification but, given postoperative treatment selection, do not establish that chemotherapy offsets the prognostic association of severe complications (Figure 2).

**Figure 2:**
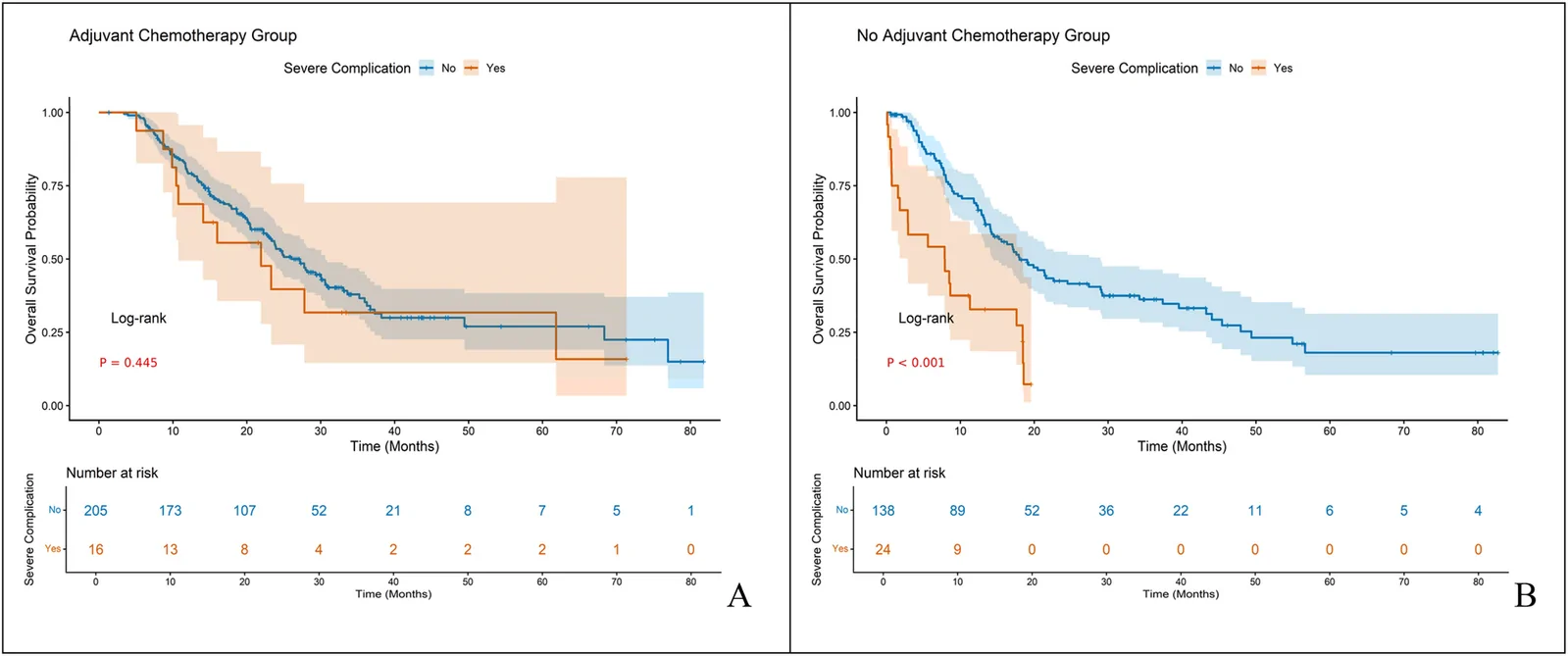
Kaplan–Meier survival curves for severe complications stratified by adjuvant chemotherapy status.

**Figure 3:**
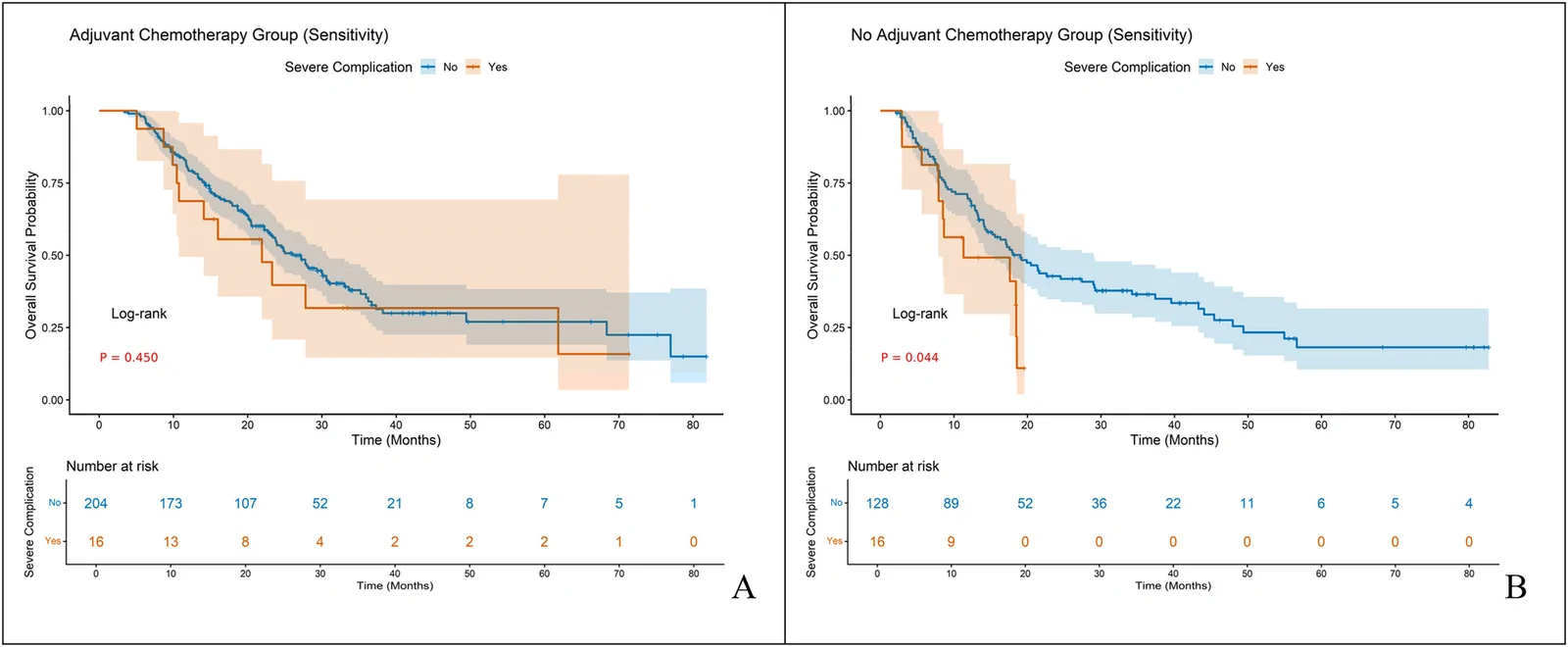
Kaplan–Meier survival curves for severe complications stratified by adjuvant chemotherapy status (excluding OS < 60 days).

**Figure 4:**
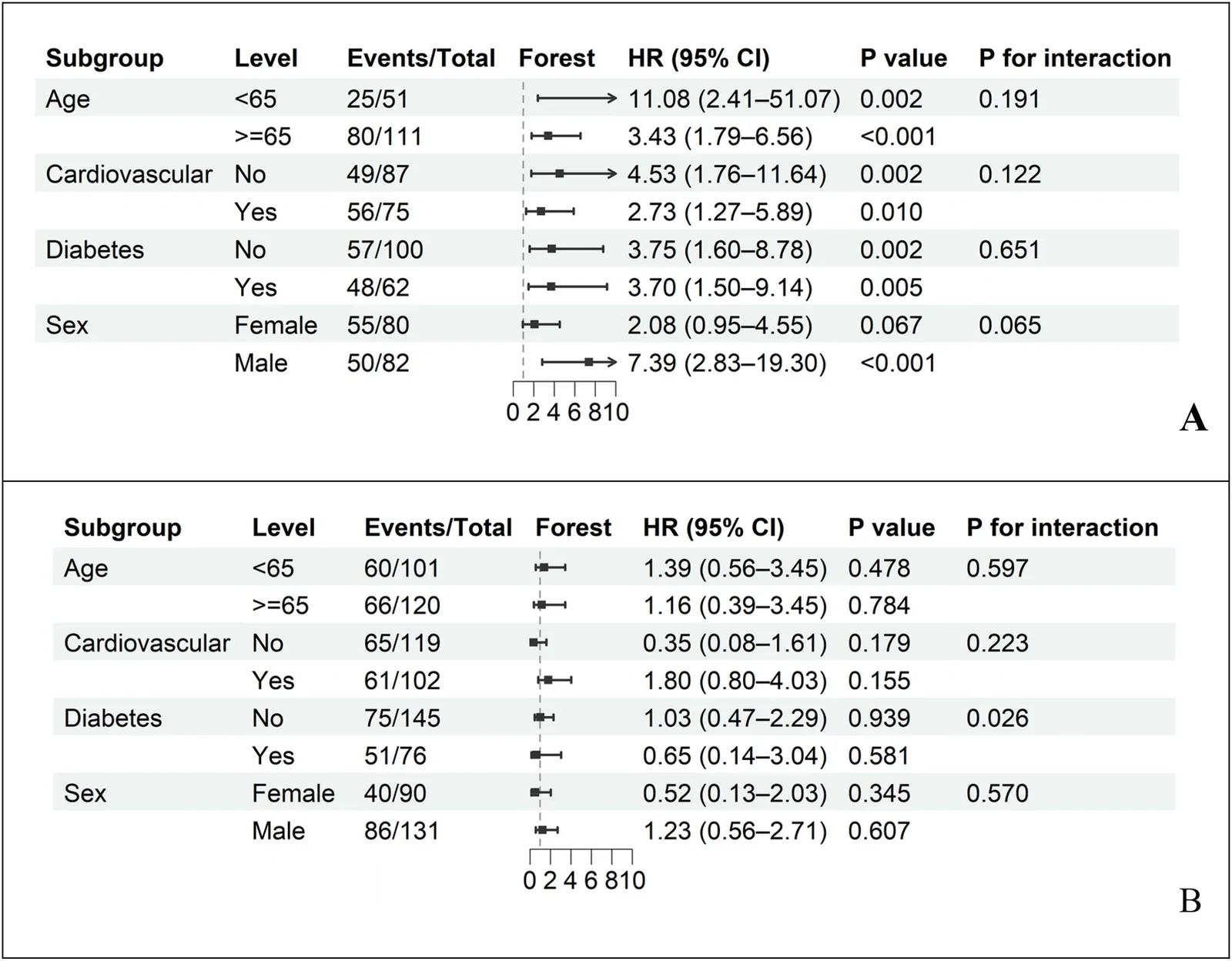
Subgroup analysis stratified by chemotherapy status.

### 3.3 Cox regression analysis

Multivariable analysis (Table 2) identified the following independent predictors of OS: severe complications (HR: 2.30, 95% CI: 1.55–3.41, *P* < 0.001), adjuvant chemotherapy (HR: 0.66, 95% CI: 0.50–0.88, *P* = 0.004), male sex (HR: 1.47, 95% CI: 1.13–1.93, *P* = 0.005), R1 margin status (HR: 1.65, 95% CI: 1.15–2.37, *P* = 0.007), lymph node ratio (HR: 2.04, 95% CI: 1.07–3.89, *P* = 0.031), and tumor size (HR: 1.28, 95% CI: 1.17–1.39, *P* < 0.001). Age was statistically significant in univariable analysis (*P* = 0.033) but lost significance in the multivariable model (*P* = 0.310).

**Table 2:** Cox regression analysis of factors affecting OS.

Variable	Univariable HR (95% CI)	*P* value	Multivariable adjusted HR (95% CI)	*P* value
Sex (Male vs. Female)	1.33 (1.02–1.73)	0.035	1.47 (1.13–1.93)	0.005
Severe complications (Yes vs. No)	2.12 (1.45–3.09)	<0.001	2.30 (1.55–3.41)	<0.001
Adjuvant chemotherapy (Yes vs. No)	0.68 (0.53–0.89)	0.004	0.66 (0.50–0.88)	0.004
Differentiation				
Middle vs. High-Middle	0.92 (0.29–2.92)	0.883	0.79 (0.24–2.56)	0.692
Middle-Low vs. High-Middle	1.06 (0.34–3.33)	0.923	0.77 (0.24–2.47)	0.662
Low-Middle vs. High-Middle	1.44 (0.43–4.88)	0.555	1.33 (0.38–4.63)	0.651
Low vs. High-Middle	1.61 (0.48–5.43)	0.438	1.29 (0.38–4.43)	0.682
Margin status (R1 vs. R0)	1.54 (1.08–2.19)	0.018	1.65 (1.15–2.37)	0.007
Vascular invasion (Yes vs. No)	1.19 (0.87–1.62)	0.270	0.96 (0.69–1.34)	0.815
Perineural invasion (Yes vs. No)	1.25 (0.94–1.66)	0.131	1.33 (0.98–1.80)	0.064
Age	1.02 (1.00–1.03)	0.033	1.01 (0.99–1.03)	0.310
BMI	0.99 (0.95–1.03)	0.674	0.96 (0.92–1.01)	0.083
LNR	1.90 (1.07–3.36)	0.028	2.04 (1.07–3.89)	0.031
Tumor size	1.26 (1.16–1.36)	<0.001	1.28 (1.17–1.39)	<0.001

The multivariable model was adjusted for age, sex, BMI, tumor differentiation, margin status, vascular invasion, perineural invasion, lymph node ratio, and tumor size. For continuous variables, HRs represent the effect per one-unit increase.

### 3.4 Interaction analysis

To evaluate whether severe-complication status modified the association between adjuvant chemotherapy and OS, we constructed a Cox regression model including a product interaction term. The results (Table 3) showed a significant interaction (interaction HR: 0.30, 95% CI: 0.14–0.64, *P* = 0.004). In the no-chemotherapy stratum, severe complications were associated with higher mortality (HR: 4.18, 95% CI: 2.49–7.01, *P* < 0.001). In the chemotherapy stratum, the association was not statistically significant (HR: 1.24, 95% CI: 0.66–2.34, *P* = 0.511). Assessing chemotherapy by complication status, the interaction model estimated HR: 0.78 (95% CI: 0.57–1.05, *P* = 0.102) in the non-severe-complication stratum and HR: 0.23 (95% CI: 0.11–0.50, *P* < 0.001) in the severe-complication stratum. These conditional estimates describe associations within one joint model and do not establish that chemotherapy caused the observed differences.

**Table 3:** Interaction effect model analysis results.

Contrast from adjusted interaction model	HR (95% CI)	*P* value
No adjuvant chemotherapy: severe vs. no severe	4.18 (2.49–7.01)	<0.001
Adjuvant chemotherapy: severe vs. no severe	1.24 (0.66–2.34)	0.511
No severe complications: Adjuvant chemotherapy vs. no Adjuvant chemotherapy	0.78 (0.57–1.05)	0.102
Severe complications: Adjuvant chemotherapy vs. no Adjuvant chemotherapy	0.23 (0.11–0.50)	<0.001
Interaction term: severe complication × adjuvant chemotherapy	0.30 (0.14–0.64)	0.004

All contrasts were derived from a Cox model adjusted for age, sex, BMI, tumor differentiation, margin status, vascular invasion, perineural invasion, lymph node ratio, and tumor size. The interaction HR is the multiplicative change in the chemotherapy association associated with severe complications.

### 3.5 Stratified cox regression analysis

Table 4 presents adjusted estimates from separate stratified Cox models using the same covariates as the main multivariable model. In the non-severe-complication group, adjuvant chemotherapy was not statistically significantly associated with lower mortality (HR: 0.76, 95% CI: 0.56–1.03, *P* = 0.075); the 95% CI: crossed 1. In the severe-complication group, the corresponding estimate was HR: 0.15 (95% CI: 0.04–0.57, *P* = 0.005). The HR: 0.23 in Table 3 and HR: 0.15 in Table 4 came from different model specifications. The former is the conditional chemotherapy contrast derived from the joint interaction model, whereas the latter is estimated in a separate stratified Cox model. The interaction *P* value was 0.004 throughout.

**Table 4:** Adjusted stratified cox regression for adjuvant chemotherapy.

Stratum	*N*	Adjusted HR (95% CI)	*P* value
No severe complications	343	0.76 (0.56–1.03)	0.075
Severe complications	40	0.15 (0.04–0.57)	0.005
Interaction *P* value	383	—	0.004

Each stratum-specific model was adjusted for age, sex, BMI, tumor differentiation, margin status, vascular invasion, perineural invasion, lymph node ratio, and tumor size. The interaction *P* value was obtained from the corresponding joint interaction model.

### 3.6 Sensitivity analysis

To examine the influence of early events, we performed a sensitivity analysis excluding patients with OS <60 days. Nineteen of 383 records were excluded, including 8 patients with severe complications and 11 without severe complications. One excluded patient had received chemotherapy. Compared with the 364 retained records, the excluded group had a higher prevalence of severe complications (42.1% vs. 8.8%) and lower chemotherapy receipt (5.3% vs. 60.4%), indicating that this sensitivity analysis changes the composition of the analytic cohort. In the 60-day analysis, median OS in the no-chemotherapy stratum remained shorter for patients with severe complications than for those without (11.2 vs. 16.1 months, log-rank *P* = 0.044), whereas the chemotherapy stratum remained non-significant (18.8 vs. 20.5 months, *P* = 0.450). As an additional descriptive check, excluding OS < 90 days removed 25 records and left 358 patients; the no-chemotherapy comparison remained directionally unfavorable for severe complications but was imprecise (17.6 vs. 19.2 months, *P* = 0.133). The 60-day threshold was retained as the primary sensitivity threshold because it excluded the earliest events while preserving more evaluable patients; it is not intended to replace the commonly used 90-day benchmark.

### 3.7 Exploratory recurrence-free survival analysis

RFS events occurred in 303 of 383 patients (79.1%), including recurrence or death from any cause. Median RFS was 9.4 months overall, 6.4 months in the severe-complication group, and 11.1 months in the non-severe-complication group (log-rank *P* < 0.001). In an adjusted Cox model including age, sex, BMI, tumor differentiation, margin status, vascular invasion, perineural invasion, lymph node ratio, and tumor size, severe complications were associated with shorter RFS (HR: 1.58, 95% CI: 1.09–2.28, *P* = 0.016). In analyses stratified by chemotherapy status, the severe-vs.-non-severe RFS comparison was not statistically significant among patients who received chemotherapy (median 7.0 vs. 10.4 months, *P* = 0.334), but remained significant among those who did not (3.7 vs. 13.1 months, *P* < 0.001) (Figure 3).

### 3.8 Subgroup analysis

To evaluate the consistency of the prognostic effect of severe complications across clinical subgroups, we performed subgroup analyses based on age, history of cardiovascular disease, history of diabetes, and sex. Among patients who received adjuvant chemotherapy, the HR for severe complications did not reach statistical significance in any subgroup, with all interaction *P* values exceeding 0.05 except for the diabetes subgroup (*P* = 0.026). Among patients who did not receive chemotherapy, the point estimates for severe complications were greater than 1 across all examined subgroups (HR range 2.08–11.08). These subgroup estimates were exploratory and imprecise because of the small severe-complication subgroup (Figure 4).

## 4 Discussion

This retrospective study evaluated whether the association between severe postoperative complications and OS differed by adjuvant chemotherapy receipt after upfront PDAC resection. The adjusted interaction test was statistically significant (interaction *P* = 0.004); however, the severe-complication subgroup was small and chemotherapy was initiated after postoperative recovery. The observed interaction therefore cannot establish that chemotherapy offsets the adverse association of severe complications and should be regarded as hypothesis-generating.

The central finding of this study is that the association between severe complications and OS differed according to chemotherapy status. Among patients who did not receive chemotherapy, severe complications were associated with a more than fourfold higher hazard of death (HR: 4.18, 95% CI: 2.49–7.01). Among those who received chemotherapy, the corresponding association was not statistically significant (HR: 1.24, 95% CI: 0.66–2.34, *P* = 0.511). These results are compatible with effect modification, but the severe-complication subgroup included only 40 patients and only 16 received chemotherapy. Treatment was administered after surgery, so patients who received chemotherapy may have been sufficiently fit to recover from complications and start treatment. The observed interaction may therefore reflect selection, confounding by postoperative fitness, or survivor bias in addition to any treatment-related association. The sensitivity analyses showed that the estimated contrast was sensitive to the early-event threshold, which further supports cautious interpretation.

Compared with the existing literature, this study provides a hypothesis-generating analysis of the association between severe postoperative complications and adjuvant chemotherapy in upfront-resected PDAC (11–15, 19, 20). Merkow et al. (18) demonstrated that postoperative complications reduce the likelihood of receiving adjuvant chemotherapy, consistent with our observation that the chemotherapy rate was lower in the severe-complication group than in the non-severe-complication group (40.0% vs. 59.8%, *P* = 0.026). Perri et al. (22) found that complications after upfront pancreatectomy with venous resection did not compromise adjuvant chemotherapy delivery or survival. Cheung et al. (21) confirmed the association between complications and receipt of adjuvant chemotherapy but did not evaluate whether chemotherapy modifies the prognostic effect of complications. Our analysis adds an interaction model, but the findings require validation in larger cohorts with detailed treatment and postoperative recovery data.

The biological mechanisms underlying the observed associations remain uncertain. Prior work in PDAC has linked surgery-induced MDSCs and the NOX2/ROS axis to postoperative outcomes (17). However, the present dataset did not include immune biomarkers or longitudinal inflammatory measurements, so an inflammation-mediated mechanism cannot be established here.

Peripheral inflammatory and nutritional indices may help characterize postoperative risk, but these measurements were not available in our institutional database and were not evaluated in this study. Their potential role should be tested in future prospective cohorts with serial sampling.

The findings should be interpreted as hypothesis-generating rather than practice-changing. Our data do not establish that adjuvant chemotherapy offsets the adverse prognostic association of severe complications or support treatment decisions based solely on complication status. Treatment decisions should incorporate postoperative recovery, performance status, organ function, nutritional status, patient preference, and the expected toxicity of the selected regimen. Timely recognition and management of complications may also affect the opportunity to deliver adjuvant therapy (20, 23–25), as examined in the PORSCH study (26), but this possible care-process pathway was not tested in the present cohort and requires prospective evaluation.

This study has several limitations. First, the single-center retrospective design is subject to selection, information, and confounding-by-indication bias. Patients who received chemotherapy had to recover sufficiently to start treatment, so the observed association with lower mortality may partly reflect postoperative fitness, survivor bias, or immortal-time bias related to post-surgical treatment initiation. Follow-up may also be less mature for patients treated late in the accrual period; follow-up in our database extended through March 2026. Second, the sample size was limited, with only 40 patients in the severe-complication group and 16 receiving chemotherapy, resulting in wide confidence intervals and limited power for interaction and subgroup analyses. Third, although our database contained free-text treatment sequences, first-chemotherapy date, and interval for all 221 chemotherapy recipients, it did not contain a standardized, time-anchored record of completed adjuvant cycles or dose intensity. It also did not record reasons for non-receipt of chemotherapy among the 162 untreated patients. Regimen-specific effectiveness and treatment-delivery analyses therefore remain limited. Fourth, our database did not contain performance status, detailed postoperative recovery measures, cause of death, or a separate historical screening log. Fifth, the 60-day sensitivity analysis changed the cohort composition, and the 90-day descriptive analysis was less precise, so neither resolves early-event or selection bias. Sixth, laboratory biomarkers such as CA19–9, NLR, PLR, SII, and PNI were not available. Seventh, the Clavien-Dindo grade ≥ 3 classification does not capture the biological heterogeneity of different complication types. Finally, RFS was analyzed as an exploratory composite outcome of recurrence or death, whereas cancer-specific survival and cause-specific mortality could not be assessed because cause-of-death data were unavailable.

## 5 Conclusion

In this retrospective cohort of 383 patients undergoing upfront curative-intent PDAC resection, severe postoperative complications were associated with shorter OS among patients who did not receive adjuvant chemotherapy, whereas the association was not statistically significant among those who received chemotherapy. The interaction between complications and chemotherapy was statistically significant, but the severe-complication subgroup was small, chemotherapy receipt was likely influenced by postoperative fitness, and estimates were sensitive to early-event exclusions. The findings are therefore hypothesis-generating. Larger prospective or multicenter studies with regimen, treatment-delivery, postoperative recovery, and cause-of-death data are needed before the results can inform treatment policy.

## Data Availability

The data analyzed in this study is subject to the following licenses/restrictions: Data for the current study are available from the corresponding author upon reasonable request. Requests to access these datasets should be directed to wushiwei202404@163.com.
